# Biomedical waste management practice in dentistry

**DOI:** 10.6026/97320630016958

**Published:** 2020-11-30

**Authors:** Aravind Kumar Subramanian, B Nivethigaa, M Srirengalakshmi, Remmiya Mary Varghese, R Navaneethan, Harish Babu

**Affiliations:** 1 Department of Orthodontics and Dentofacial Orthopedics, Saveetha Dental College, Saveetha institute of Medical and Technical Sciences, Saveetha University, Chennai, Tamilnadu, India

**Keywords:** Bio dental waste, biomedical waste, green dentistry, biohazards, waste management

## Abstract

Biomedical Waste Management practice in Dentistry is an important issue. Therefore, it is of interest to document awareness on such issues among clinical practitioners, academicians and students. A survey was completed using a questionnaire from 355 dentists consisting of 201 students, 39 academicians and 115 clinicians in India. Analysis of the survey data shows that majority of students, practitioners, and academicians are aware of laws binding with such issues. However, the Biomedical Waste Management practice among them is not satisfactory. Therefore, education on such issues among clinical practitioners, academicians and students is critical in this part of the globe.

## Background

Medical waste, household waste, industrial plastics, and e-waste are the primary cause of global pollution of air, water and soil, which can adversely affect the biosystem and life in our planet [1-3]. The effects of pollution don't stop with just befouling the surfaces, rather they go aboard with steep raise in the temperature as in global warming which leads to depletion of the protective ozone envelope. These further cause extinction of marine micro and macro flora, thawing effect on the ice resulting in the all-embracing detrimental consequence to the entire ecosystem [4,^5^]. Bio Medical Waste (BMW) includes scrap from any procedure that deals with a living organism like preventive care involving immunization or any particulate material utilized in the diagnosis and treatment process, laboratory specimen, animal samples used in testing, and blood bank utilities [6]. Bio medical waste should be disposed with caution [7]. Many modifications had been implemented to improvise the methods of biowaste segregation and disposal [8]. Improper disposal of hazardous waste into the common sites lead to deleterious effects among the sanitation workers [9]. Several outbreaks associated with transmission of diseases like Hepatitis through syringes have been documented in the last few decades due to this menace [10]. Therefore, it is of interest to document awareness on such issues among clinical practitioners, academicians and students.

## Materials and Methods:

This questionnaire-based study was cross-sectional and performed by four investigators. Existing literature sources were searched for data on biomedical waste, protocols of management, and hazards they pose to the environment. A preliminary questionnaire was constructed after discussion and resolution with all the four investigators. This comprised of three categories of dental personnel. Data for the study was collected through an online survey platform (Google Forms). Online data collection eliminated possible human errors which otherwise could occur during the transfer of paper work or any altered responses similar to those seen during personal interviews, thus overcoming bias with regard to data collection and evaluation. The survey data was collected from 355 dentists consisting of 201 students, 39 academicians, and 115 clinicians with a private dental practice.

## Survey Link:

https://docs.google.com/forms/d/e/1FAIpQLScqo2lceJCc4DqY4YZ48jcLLWrXp_XJv5hhaKv65gejOMUZVA/viewform?usp=sf_link

## Structure of the questionnaire:

The questionnaire consisted of few questions related to the demographics, including the geographical location of the participant. In order to avoid conflicts regarding the nature of problems, the questionnaire was divided into three sections, which included separate questions for three groups. Group A consisted of undergraduate and postgraduate students pursuing dentistry and dental specialties in various regions across the country. Group B consisted of academician working in various dentistry teaching institutions across the country. Group C consisted of clinicians (private practitioners) who manage private dental clinics.

## Validation of the questionnaire:

The investigators and other dental practitioners did validation of the questionnaire and changes were made accordingly. The final validated questionnaire was shared online over various social media platforms.

## Survey location:

Dental practitioners and dental students who had performed clinical dental procedures across the country were allowed to participate in the study. This would allow for a wide knowledge about students' and practitioners' understanding of the environmental hazards of healthcare waste and the precautions and guidelines with which management of waste has been taught and practiced across various states.

## Analysis of the data:

The contents were transferred to an excel sheet where further coding was done. The excel was analyzed by two examiners separately and the final values were crosschecked to reduce error. Data was analyzed using the SPSS (version 26.0) software.

## Statistical analysis:

Frequency distribution for the study subjects was calculated based on the nature of the hierarchical work position i.e., Student, Practitioner, and Academician and also among the practitioners based on the number of years of experience. For all other survey questions, Chi-square test was performed along with the frequency distribution of individual responses. All tests were two sided and p-value of 0.05 and lesser was considered to be statistically significant and anything more than 0.05 was considered to have no statistical significance.

## Results and Discussion:

The results can be categorized as areas where the dentists and the dental students were well aware, moderately aware and least aware. There was a wide spread awareness amongst the participants about the laws & rules on bio medical waste management, color coded segregation and with the usage of hazardous symbols. According to the "Safe management of healthcare waste" rules provided by the WHO, there is a need for segregation of biomedical waste at the point of waste generation to reducee the amount of the risk faced by the sanitation workers [11] . The present study shows that 81.6% of the students, 88.7% of the practitioners, and 76.9% of the academicians were familiar with the laws and rules on biomedical waste management and methods of segregation before disposal. It is a well-known fact that the Dental healthcare workers are also more prone for droplet spread of the disease [12] and should also be immunized for Hepatitis B. In the present study, more than three-fourth of the dentists have trained their dental assistants regarding risk of handling biomedical waste and the importance of vaccination [13,14]. Following the present outbreak of COVID-19, special rules have been put forth by the National Pollution Control Board along with the health ministry to prevent community spread of the disease from affected individuals, protect the soil, air, and water from being infected, and protect the community healthcare and sanitation workers from acquiring the disease during the process of handling waste disposal [15]. In the present study 93% of the students, 77.4% of the practitioners, and 87.2% of the academicians followed specialized waste disposal protocol. In segregation of wastes, anatomical waste from human tissues and animal waste are segregated in yellow colored non-chlorinated bags and disposed by deep burial in non-residential areas, incineration or plasma pyrolysis. Almost all of the dental professionals (93% students, all practitioners, and 97.4% academicians) practice disposal with separate color-coded bins. The system of symbolic representation for biomedical waste provides ease in identifying the nature of the waste [11]. In the present study, 77.15% of the students, 89.6% of the practitioners, and 74.4% of the academicians had a knowledge of the symbols used to represent biohazardous waste. (Table 1 - see PDF)

We found a moderate level of awareness regarding the hazardous effects of the wastes generated. Although the Impression materials and gypsum-based products are non-hazardous in dry form, they become hazardous when there is water sorption and releases harmful microorganism and gases like hydrogen sulphides that has a bad odor. We found that 62.75% of the students, 54.8% of the practitioners, and 76.9% of the academicians consider impression material to be hazardous and dispose them in yellow bags. Another hazardous material in our practice is the Silver ions. These ions in the free form have properties of undesirable interactions with other components in the drainage. Fixer, Developer solution (contaminated with fixer), undeveloped X-ray film contain considerable amount of silver ions. Cleaner solution contains chromium. All these are categorized as hazardous waste [16].In the current study, only 33.3% of the students, 46.1% of the practitioners, and 28.2% of the academicians were aware of the hazardous nature of the fixer solution (Table 2 - see PDF).

There was a very low level of awareness in dealing with sharp wastes, amalgam recycling, chemical waste management and incineration procedure. Ideally the waste consisting of needles used in syringes, glasses, scalpel etc. should be disinfected by autoclaving or any other chemical treatment, followed by mutilation and shredding. Less than 15% of the total respondents follow the ideal method of disposal of sharps and 28.4% of the dentists in all groups mix blood-soaked waste with sharps during disposal. The usage of amalgam in our practice is controversial as Mercury in blood and urine have been detected and reported. When we are disposing Amalgam restored teeth using incineration technique, it releases harmful mercury vapor. This should be avoided and disposed to amalgam recycler [17,18]. In the present study, considerable usage of dental amalgam was seen whereas only 7.5% of the students, 3.5% of the practitioners, and 2.6% of the academicians dispose it after chemical disinfection in recycler. Waste management includes pharmaceutical waste, chemical waste and bio medical waste. Pharmaceutical waste consists of expired medicines or other pharmaceutical products, which can lead to the development of resistant microorganism in the area of disposal. Hence should be handed over to the concerned manufacturer in black colored bags. Chemical waste in any form may be toxic with corrosion potential, combustible, oxidizing nature, and reactive on surface. [19]. Solid waste can be buried in deep landfills in yellow color-coded bags and liquids can be disposed in the drainage after required preprocessing. Dentists lack awareness of this aspect of managing chemical waste or execution of proper protocol. The biomedical waste should be stored at room temperature and can lead to increase in microbial load and harmful chemical substrates by putrefaction [11]. Hence it should be transported within 24 hours. Almost 34.3% of the students, 46.2% of the academicians, and 5.2% of the practitioners are not sure about the temperature range. Nearly 28.4% of the students, 20.9% of the practitioners and 17.9% of the academicians are clear about retaining the waste for not more than 24 hours, if they weren't refrigerated. The wastes are also disposed using incineration and non-incineration methods. Incineration is a technique where the scrap is combusted at high temperature resulting in the formation of small particulate material. This poses disadvantage of releasing harmful gaseous materials, which attenuate the air pollution. Nearly half of the respondents in all categories incinerate the waste generated. 32.8% of the students, 98.3% of the practitioners, and 20.5% of the academicians still use chlorinated bags for disposal in their workplace, while being unaware of adverse effects. The non-incineration methods of waste disposal include thermal, chemical, irradiative, biological and mechanical process. In the present study, majority of the dentists used thermal process like autoclaving and a very few used chemical processes like use of hypochlorite (Table 3 - see PDF)

## Conclusion

Analysis of the survey data shows that majority of students, practitioners, and academicians are aware of laws binding with such issues. However, the Biomedical Waste Management practice among them is not satisfactory. Therefore, education on such issues among clinical practitioners, academicians and students is critical in this part of the globe.
